# Exploration into biomarker potential of region-specific brain gene co-expression networks

**DOI:** 10.1038/s41598-020-73611-1

**Published:** 2020-10-13

**Authors:** Yuqing Hang, Mohammed Aburidi, Benafsh Husain, Allison R. Hickman, William L. Poehlman, F. Alex Feltus

**Affiliations:** 1 Department of Genetics and Biochemistry, Clemson University, Clemson, 29634 USA; 2 Biomedical Data Science and Informatics Program, Clemson University, Clemson, 29634 USA; 3 Center for Human Genetics, Clemson University, Clemson, 29634 USA

**Keywords:** Genome informatics, Machine learning, Gene expression, Genetic interaction, Genomics

## Abstract

The human brain is a complex organ that consists of several regions each with a unique gene expression pattern. Our intent in this study was to construct a gene co-expression network (GCN) for the normal brain using RNA expression profiles from the Genotype-Tissue Expression (GTEx) project. The brain GCN contains gene correlation relationships that are broadly present in the brain or specific to thirteen brain regions, which we later combined into six overarching brain mini-GCNs based on the brain’s structure. Using the expression profiles of brain region-specific GCN edges, we determined how well the brain region samples could be discriminated from each other, visually with t-SNE plots or quantitatively with the Gene Oracle deep learning classifier. Next, we tested these gene sets on their relevance to human tumors of brain and non-brain origin. Interestingly, we found that genes in the six brain mini-GCNs showed markedly higher mutation rates in tumors relative to matched sets of random genes. Further, we found that cortex genes subdivided Head and Neck Squamous Cell Carcinoma (HNSC) tumors and Pheochromocytoma and Paraganglioma (PCPG) tumors into distinct groups. The brain GCN and mini-GCNs are useful resources for the classification of brain regions and identification of biomarker genes for brain related phenotypes.

## Introduction

The human brain is a complex system encompassing countless cells that coalesce into hundreds of different regions and patterns of functional connectivity^1^. The coherence between brain regions results in canonical functions like vision, language, and memory^2^. The complexity of the brain is mainly due to the spatial and temporal alteration of large amounts of gene expression during developmental specification and maturation^3^. However, the region-specific description and control of gene expression patterns across the human brain has yet to be fully revealed.

Fortunately, recent high-resolution genome wide transcriptome profiling studies provide deeper insight into brain gene expression, especially in the context of disease-associated expression shifts in different brain regions. For example, Twine et al.^4^ studied transcriptome profiles from both healthy brains and brains from patients with Alzheimer’s disease (AD). They found significant differences in gene expression levels, splicing isoforms, and alternative transcription start sites between healthy and AD brains. Another group created a gene expression model based on transcriptome datasets of the healthy human brain from the Allen Brain Atlas. This model can be used to identify potentially new candidate genes implicated in neurological diseases using machine learning^5,6^. Other studies include identifying gene expression patterns related to developmental origin of brain regions, brain functions, and brain-specific diseases like autism^7–9^. A brain transcriptome resource we leveraged in this study is from the Genotype-Tissue Expression (GTEx) project^10^ that characterized 54 tissues from 948 human donors. Thirteen of those tissue were from specific regions of the human brain.

With the increasing number and diversity of high-resolution human brain gene expression datasets, it is becoming easier to detect polygenic *biomarker systems *relevant to a specific medical condition or brain region. For example, using pairwise gene expression correlation tests across genes in a gene expression matrix (GEM), a gene co-expression network (GCN) can be constructed and utilized to detect co-functional gene sets^11–14^. In a GCN, each node represents a gene or gene product, and two nodes are connected by an edge if they have a significant co-expression relationship. A group of highly-connected genes in a GCN have a higher likelihood of being functionally related relative to a group of poorly connected genes. We construct GCNs using software called Knowledge-Independent Network Construction (KINC), which employs Gaussian Mixture Models (GMMs) to cluster samples before a pairwise correlation calculation^12^. KINC deconvolutes mixed-condition pairwise expression profiles into condition-specific edges that can be merged into a condition specific GCN.

Condition-specific GCNs encompass candidate biomarker systems relevant to that condition. The effectiveness of the biomarkers to discriminate conditions can be formally tested using machine learning and other classification techniques. For example, Gene Oracle is a software package that implements a deep learning model to classify biological samples using gene expression features as input^15^. In the Gene Oracle algorithm, expression profiles of candidate gene sets are tested for significant non-random classification potential of sample types (i.e. classification labels). The gene sets are then decomposed into the most discriminatory candidate biomarker gene sets. In this approach, the relevance of a biomarker system is formally quantified and refined to the core biomarker features.

In this study, our goal was to identify condition-specific GCNs for normal human brain regions. Towards this goal, we constructed a brain GCN using a GEM derived from 13 brain RNA-seq datasets obtained from the GTEx project^10^. We deconvoluted the brain edges into brain region mini-GCNs and characterized highly interconnected genes. We then used Gene Oracle to classify the input brain samples with these mini-GCNs to test their biomarker potential on normal brain regions. Finally, we tested if the brain region-specific genes tumor expression profiles were able to discriminate the brain from non-brain human tumors.

**Figure 1:**
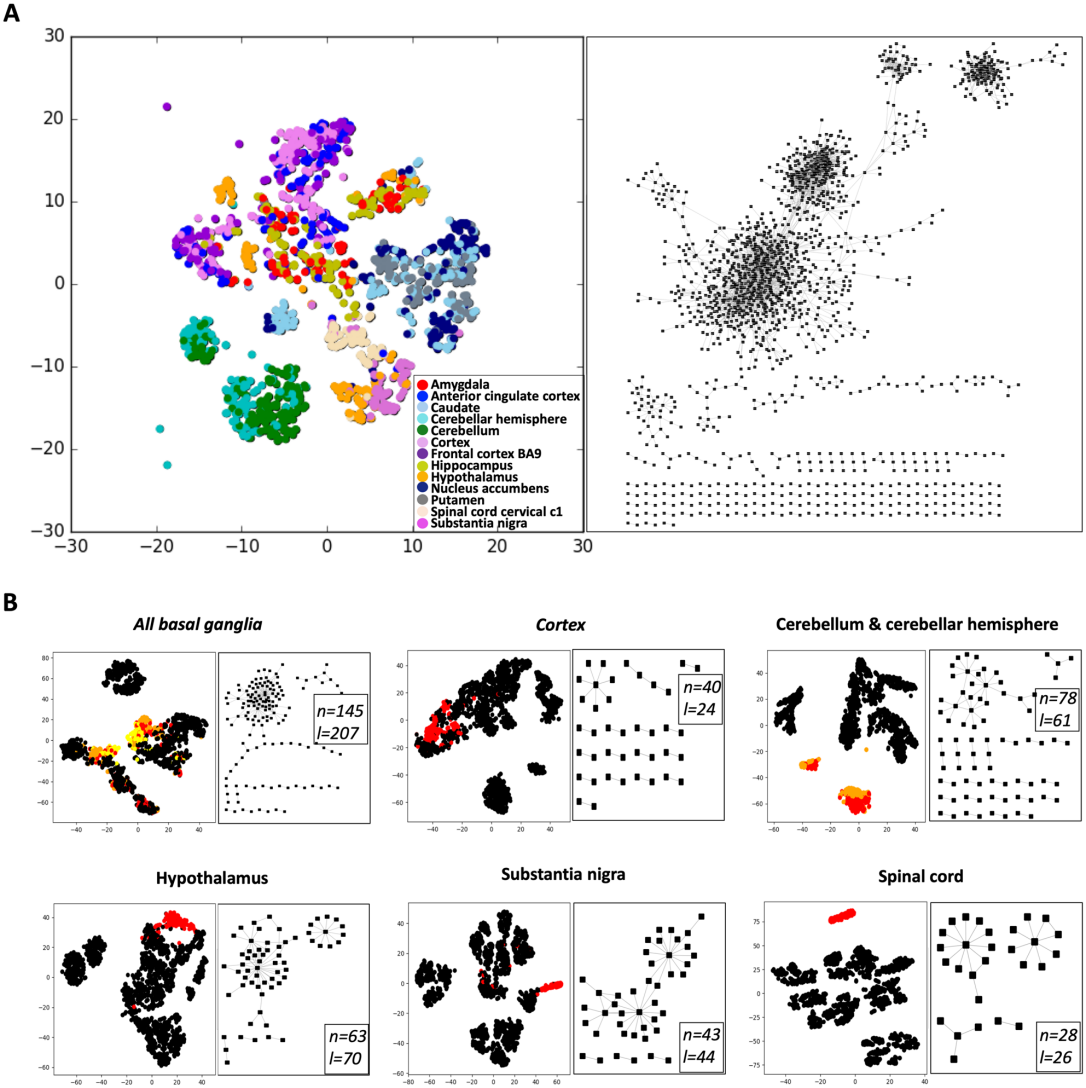
Normal brain gene co-expression network. (**A**) The right panel represents the whole gene co-expression network (GCN) constructed from 1671 GTEx brain RNAseq samples from 13 different brain regions. The left panel is the corresponding t-SNE visualization for the 1691 brain GCN genes where RNA expression profiles sorted regions into multiple clusters. Each color represents a different region shown in the legend. (**B**) Six brain region mini-GCNs are shown on the right side of each panel. Corresponding t-SNE visualization pictures for those region-specific genes are shown on the left side of each panel. Non-black dots in each tSNE plot represent the corresponding region-specific samples and black dots represent samples from all other regions. For all basal ganglia specific gene sets, red, orange and yellow dots represent caudate basal ganglia, nucleus accumben basal ganglia, and putamen basal ganglia samples respectively. The red and orange dots from cerebellum and cerebellar hemisphere specific gene sets represent cerebellum and cerebellar hemisphere samples respectively. All red dots from other region-specific gene sets represent the particular region-specific samples.

## Results

### Brain region-specific gene co-expression network (GCN) construction

We wanted to identify region-specific gene co-expression patterns in the brain using KINC^13^. To do this, we analyzed 1671 GTEx gene expression profiles from 56,202 genes across 13 different brain regions. Prior to GCN construction, the GTEx GEM was preprocessed by log2 transformation of the expression values, applying the Kolmogorov–Smirnov test to remove outlier distributions, and performing quantile normalization on the GEM. The full brain GCN contains 1691 nodes and 7812 edges (Supplemental Table 1; Fig. 1A—right panel). The GCN was further dissected into 183 linked community modules (LCMs)^16^.

We then performed sample label enrichment for all modules and edges in the brain GCN (Supplemental Tables 2 and 3). In the sample label enrichment for modules, we considered to use the p-value threshold of 1E−3 for significantly enriched modules. Because modules contain different numbers of genes, there are very few modules that are enriched significantly in only one brain region. Thus, we calculated the sample label enrichment for each edge. The edges with a p-value less than 1E−10 were considered to be significantly enriched because this p-value is close to maximizing the number of edges and nodes. The number of enriched edges for each specific region with the adjusted p-value less than 1E−3, 1E−5, 1E−15, and 1E−20 were also collected separately and the tSNE visualization was ran for each of those gene sets (Supplemental Table 4 and Supplemental Figure 1). One can see from the results that even though the threshold p-value of 1E−15 had more edges and nodes in total, most regions had a decrease in the number of specific edges. For example, the number of edges and nodes in the basal ganglia, cerebellum, and spinal cord regions was lower when the threshold was 1E−15 compared to a p-value threshold of 1E−10. Exceptions to this included the cortex specific edges, which increased slightly in number, and the hypothalamus where the number of specific edges increased strikingly. Furthermore, the tSNE plots showed that the region-specific genes cannot separate samples very well for p-value thresholds of 1E−3 and 1E−5, while they can for thresholds of 1E−10, 1E−15, and 1E−20. Thus, we used a p-value of 1E−10 as the threshold of significance for the sample label enrichment for edges.

The identified region-specific sub-GCNs with adjusted p-value less than 1E−10 are shown in Fig. 1B—right panel for each region. Global attributes for both full brain GCN and region sub-GCNs are shown in Table 1. For example, the 160 brain caudate (basal ganglia) samples significantly contributed to 2076 edges (p < 1E−10) that contained 690 nodes and connected 131 modules. These brain caudate (basal ganglia) nodes had high connectivity (k = 6.02), and among the 690 nodes, 33,554 eQTLs were found in the GTEx database. The average gene expression values for enriched nodes ($\mu =3.89$, $\sigma =2.49$) was higher than all GTEx gene expression values ($\mu =0.57$, $\sigma =3.34$).

**Table 1:** Normal brain GCN edge attributes.

Region	Samples	Nodes	Edges^a^	Modules^a^	All genes specific eQTLs	[RNA] All genes mean	[RNA] All genes stdev	[RNA] Enriched node mean	[RNA] Enriched node stdev	k	Unique edges	Unique edge percentage
Full network (all regions)	1671	1691	7812	183	38,549	0.57	3.34	3.65	2.71	9.24	434	0.06
Brain amygdala	100	188	145	87	4,097	0.57	3.34	4.50	1.99	1.54	0	0.00
Brain anterior cingulate cortex BA24	121	419	468	41	11,833	0.57	3.34	4.62	1.90	2.23	0	0.00
Brain caudate (basal ganglia)	160	690	2076	131	33,554	0.57	3.34	3.89	2.49	6.02	200	0.10
Brain cerebellar hemisphere	136	270	225	4	43,794	0.57	3.34	4.86	1.90	1.67	1	0.00
Brain cerebellum	173	327	301	13	129,623	0.57	3.34	4.98	1.84	1.84	60	0.20
Brain cortex	158	646	928	74	42,199	0.57	3.34	4.87	1.87	2.87	24	0.03
Brain frontal cortex BA9	129	545	735	54	20,048	0.57	3.34	5.03	1.81	2.70	0	0.00
Brain hippocampus	123	377	909	114	8147	0.57	3.34	3.99	2.70	4.82	2	0.00
Brain hypothalamus	121	440	1502	103	7853	0.57	3.34	3.67	2.59	6.83	70	0.05
Brain nucleus accumbens (basal ganglia)	147	536	693	73	24,106	0.57	3.34	4.77	1.83	2.59	5	0.01
Brain putamen (basal ganglia)	124	427	466	75	14,446	0.57	3.34	4.70	1.84	2.18	2	0.00
Brain spinal cord cervical c1	91	145	95	37	14,663	0.57	3.34	3.80	2.26	1.31	26	0.27
Brain substantia nigra	88	111	84	51	2782	0.57	3.34	3.75	2.30	1.51	44	0.52

[1] For edge enrichment, we consider the significant edges for each sub-cluster as those with p-values less than 1E−10; [2] For module enrichment, we consider the significant modules for each sub-cluster will be those with p-values less than 1E−3.

**Figure 2:**
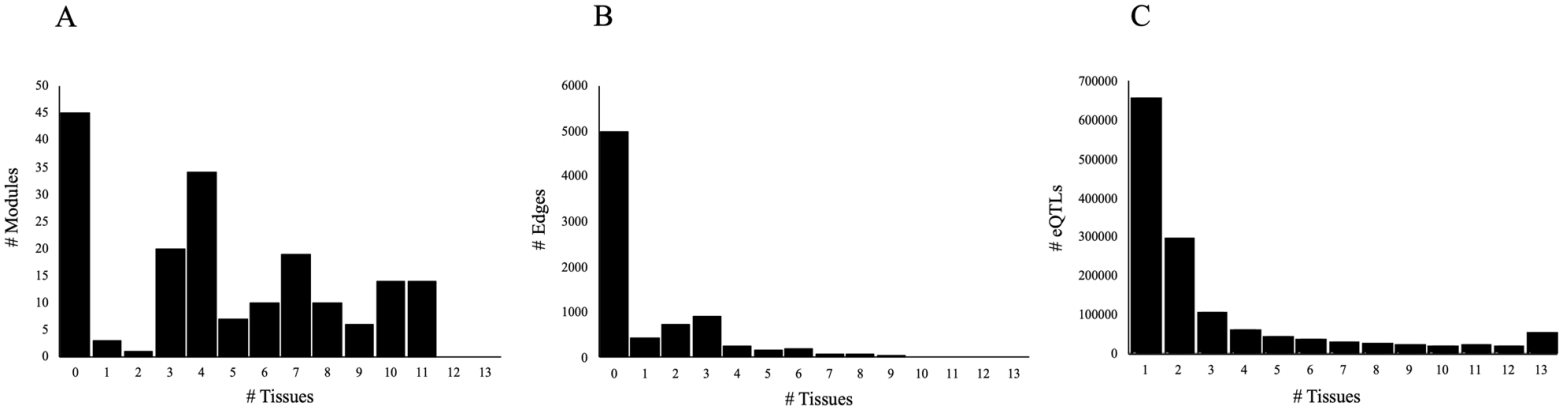
Brain region-specific GCN attributes. (**A**) Number of link community modules unique to 0–13 brain regions. (**B**) Number of edges unique in 0–13 brain regions. (**C**) Number of region-specific edge associated GTEx eQTLs unique in 1–13 brain regions.

We counted the number of modules, edges, and eQTLs that were enriched for each brain region (Fig. 2). Most modules were enriched in multiple brain regions. However, there were three modules enriched in only one specific brain region, and one more module enriched in two brain regions. Similar to the modules, the majority of the edges were not enriched in one single region, with the exception of 434 that were present in only one brain region. Most eQTLs were found in only one brain region, and the number of eQTLs decreased when there were more shared regions, except for the number of eQTLs shared by all 13 brain regions. For each region’s enriched edges, we also counted how many of them were associated with other regions (Supplemental Figure 2). Most edges were enriched in more than one brain region.

For the 434 region-specific edges, we identified edges that were unique for one region as shown in Table 2. For example, 139 nodes and 200 edges were found that are only enriched in brain caudate (basal ganglia) samples. Only 22 genes out of the 139 unique caudate genes contained eQTLs and 917 eQTLs were found in total. On average, each unique node had 41.68 eQTLs. Of the 13 brain regions, ten contained region-specific edges. According to the anatomy of the brain, we combined some of the region-specific edges together to form six region-specific edge lists. For example, the basal ganglia consists of the caudate, the nucleus accumbens, and the putamen. Therefore, we combined those region-specific edges together. The cerebellar and cerebellar hemisphere samples were taken from the same site in the brain, so we combined those two lists together. The hippocampus only contained two region-specific edges, therefore it was not large enough to construct its own sub-GCN. In total, six overarching region-specific edge lists were generated from the brain GCN. We used these new sets to construct sub-GCNs and visualize their gene expression patterns.

**Table 2:** Unique region-specific edge attributes.

Region	Edges	Nodes	eQTLs	Nodes counted	eQTLs/node
Full network (all regions)	434	344	2228	59	37.76
Brain amygdala	0	0	0	0	0
Brain anterior cingulate cortex BA24	0	0	0	0	0
Brain caudate (basal ganglia)	200	139	917	22	41.68
Brain cerebellar hemisphere	1	2	0	0	0
Brain cerebellum	60	76	874	17	51.41
Brain cortex	24	40	296	9	32.89
Brain frontal cortex BA9	0	0	0	0	0
Brain hippocampus	2	4	0	0	0
Brain hypothalamus	70	63	108	6	18
Brain nucleus accumbens (basal ganglia)	5	9	23	1	23
Brain putamen (basal ganglia)	2	4	0	0	0
Brain spinal cord cervical C1	26	28	0	0	0
Brain substantia nigra	44	43	10	4	2.5

To see how region-specific gene subsets separate the brain regions, we performed t-SNE. t-SNE is a dimensionality reduction algorithm for visualization of high dimensional data into two or three dimensions^17^. Using all the genes from the full brain GCN as input to t-SNE, the regions separated at different degrees (Fig. 1A—left panel). The main observation was the separation of the cerebellum and cerebellar hemisphere samples from other region samples. The expression pattern for other brain regions mixed together and could not be distinguished.

Using different sets of region-specific sub-GCN genes as input to t-SNE, the region distribution varied (Fig. 1B—left panel). For example, the basal ganglia region, consisting of caudate basal ganglia, nucleus accumbens basal ganglia, and putamen basal ganglia samples, did not separate basal ganglia samples from other region samples based on the expression patterns of the 145 unique basal ganglia genes. This pattern was also observed in the cortex samples when using the 40 unique cortex genes as input. However, the expression pattern for 28 spinal cord unique nodes separated the spinal cord samples from any other brain region samples. Also, cerebellum specific genes were able to separate cerebellum and cerebellar hemisphere samples from all other samples. The t-SNE visualization for each region based on its enriched nodes is shown in Supplemental Figure 3.

We performed functional enrichment analysis on full brain GCN modules (Supplemental Table 5) as well as brain region-specific nodes (Supplemental Table 6). Table 3 lists the region-specific module information, and Table 4 lists their corresponding functional enrichment results.

To determine if the region-specific edges were coding or non-coding genes, we counted the gene classes for all GTEx input genes, brain GCN genes, as well as each region’s specific gene list (Supplemental Table 7). About one third of the GTEx input genes are protein coding genes, but of those from the brain GCN and the region-specific gene lists, almost all the genes are protein coding. Interestingly, among the 1,671 genes from brain GCN, there are 39 genes that are lncRNA (Supplemental Table 8). This could be our future topic of study.

**Table 3:** Region-specific module information.

Module	Edges	Enriched region	p-value
M005	5	Cerebellum	4.20E−11
M126	3	Cerebellar hemisphere; cerebellum	2.54E−78; 1.57E−102

**Table 4:** Unique brain module functional enrichment analysis.

Module	Region	Adj. p^1^	Term ID	Term definition
M0005	Cerebellum	1.22E−03	MIM:114850	CARBOXYPEPTIDASE A1
M0005	Cerebellum	1.22E−03	MIM:246600	PANCREATIC LIPASE
M0005	Cerebellum	1.22E−03	MIM:276000	PROTEASE, SERINE, 1
M0005	Cerebellum	1.94E−03	GO:0005615	Extracellular space
M0005	Cerebellum	3.42E−03	GO:0006508	Proteolysis
M0005	Cerebellum	1.57E−03	GO:0008233	Peptidase activity
M0005	Cerebellum	5.63E−03	GO:0008236	Serine-type peptidase activity
M0005	Cerebellum	2.25E−03	GO:0016787	Hydrolase activity
M0005	Cerebellum	7.70E−03	GO:0061365	Positive regulation of triglyceride lipase activity
M0005	Cerebellum	9.55E−03	IPR001314	Peptidase S1A, chymotrypsin family
M0005	Cerebellum	8.05E−03	IPR018114	Serine proteases, trypsin family, histidine active site
M0005	Cerebellum	6.96E−03	IPR033116	Serine proteases, trypsin family, serine active site
M0005	Cerebellum	1.18E−03	PF00089	Trypsin
M0005	Cerebellum	9.42E−03	R-HSA-196854	Metabolism of vitamins and cofactors
M0126	Cerebellar hemisphere; cerebellum	2.18E−03	MIM:603140	PHOSPHATIDYLINOSITOL 5-PHOSPHATE 4-KINASE, TYPE II, ALPHA
M0126	Cerebellar hemisphere; cerebellum	2.18E−03	MIM:609410	SYNAPTOJANIN 2
M0126	Cerebellar hemisphere; cerebellum	2.18E−03	MIM:610072	ERMIN
M0126	Cerebellar hemisphere; cerebellum	2.18E−03	MIM:616027	ACTIN-BINDING PROTEIN ANILLIN
M0126	Cerebellar hemisphere; cerebellum	8.74E−03	IPR031970	Anillin, N-terminal domain
M0126	Cerebellar hemisphere; cerebellum	8.74E−03	IPR034973	Synaptojanin-2, RNA recognition motif
M0126	Cerebellar hemisphere; cerebellum	8.74E−03	IPR034974	Synaptojanin-2
M0126	Cerebellar hemisphere; cerebellum	6.71E−03	PF08174	Cell division protein anillin
M0126	Cerebellar hemisphere; cerebellum	5.03E−03	PF08952	Domain of unknown function (DUF1866)
M0126	Cerebellar hemisphere; cerebellum	3.35E−03	PF16018	Anillin N-terminus
M0126	Cerebellar hemisphere; cerebellum	4.30E−03	R-HSA-1483255	PI Metabolism
M0126	Cerebellar hemisphere; cerebellum	1.73E−03	R-HSA-1660499	Synthesis of PIPs at the plasma membrane

[1] Bonferroni adjusted p-value< 0.01.

### Brain region biomarker validation

**Figure 3:**
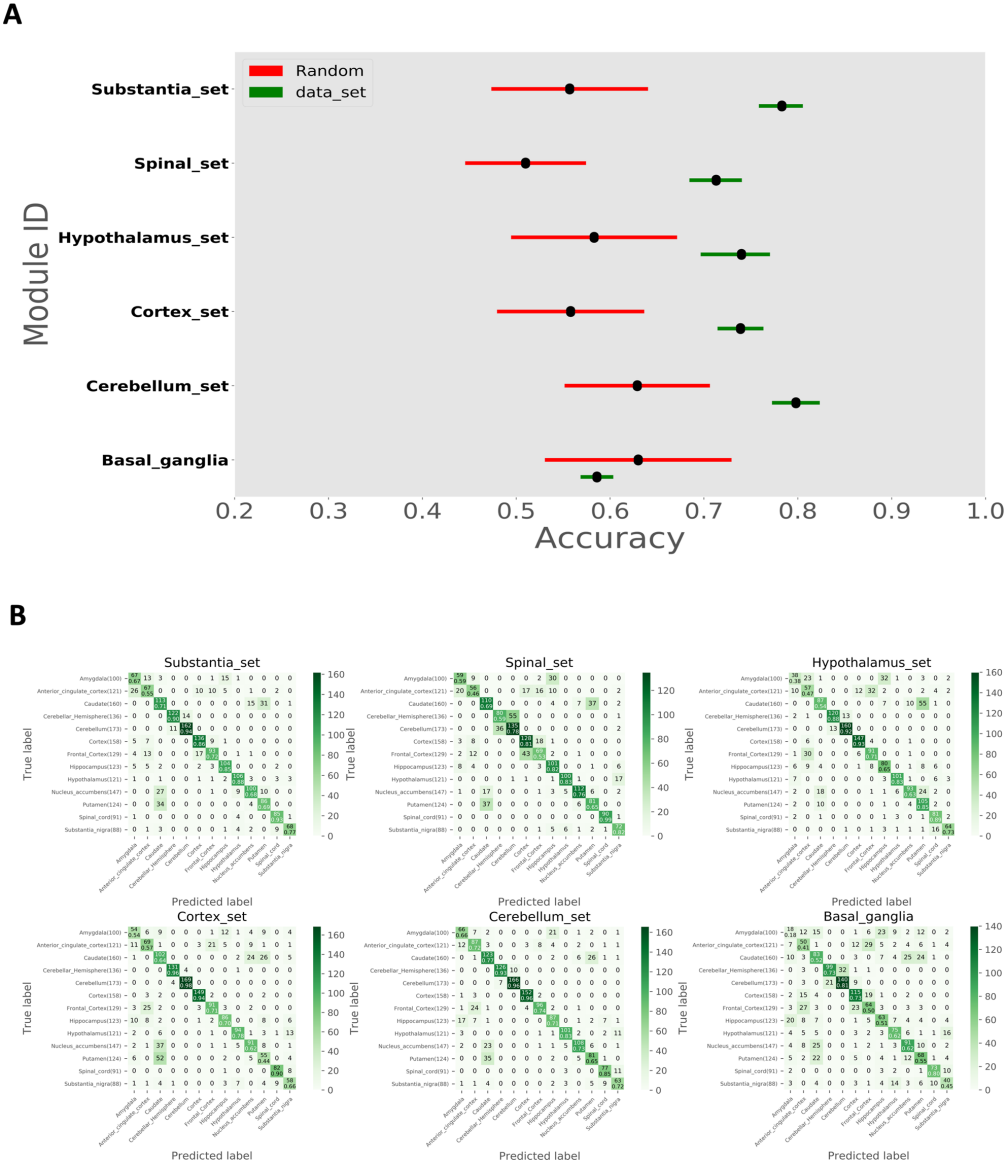
Gene Oracle classification of brain regions with brain region-specific edges. (**A**) Classification accuracy (X-axis) of region-specific gene sets (Y-axis; green bars) versus matched number of random genes (red bars) over 1671 GTEx brain samples from 13 different brain regions. (**B**) Confusion plot showing precise classifications (diagonal boxes) and misclassified samples for each region-specific gene sets. The upper number in the diagonal boxes indicates the number of samples that are correctly classified, and the lower number indicates its percent for each class. Other boxes show a number of misclassified samples.

The six brain region-specific gene sets identified by GCN analysis were evaluated using Gene Oracle software which determines classification accuracy of a set of target genes relative to an identical number of random genes. We ran Gene Oracle phase I over 1671 samples from 13 different brain regions. Genes of each region’s specific set were used as features to classify samples into 13 brain regions. The output accuracy of each region’s specific set is shown in Fig. 3A. The following five sets showed significant classification potential: substantia nigra, spinal cord, hypothalamus, cortex, and cerebellum. Surprisingly, the sixth set (basal ganglia) was not significant as random genes provided a similar accuracy. To show the precise classification and the contribution of a region-specific gene set to each class, we generated a confusion matrix for each set (Fig. 3B). Darker green correlates to a higher classification accuracy. We observed that in most cases the region-specific gene sets classified their respective regions more accurately than other sets.

**Figure 4:**
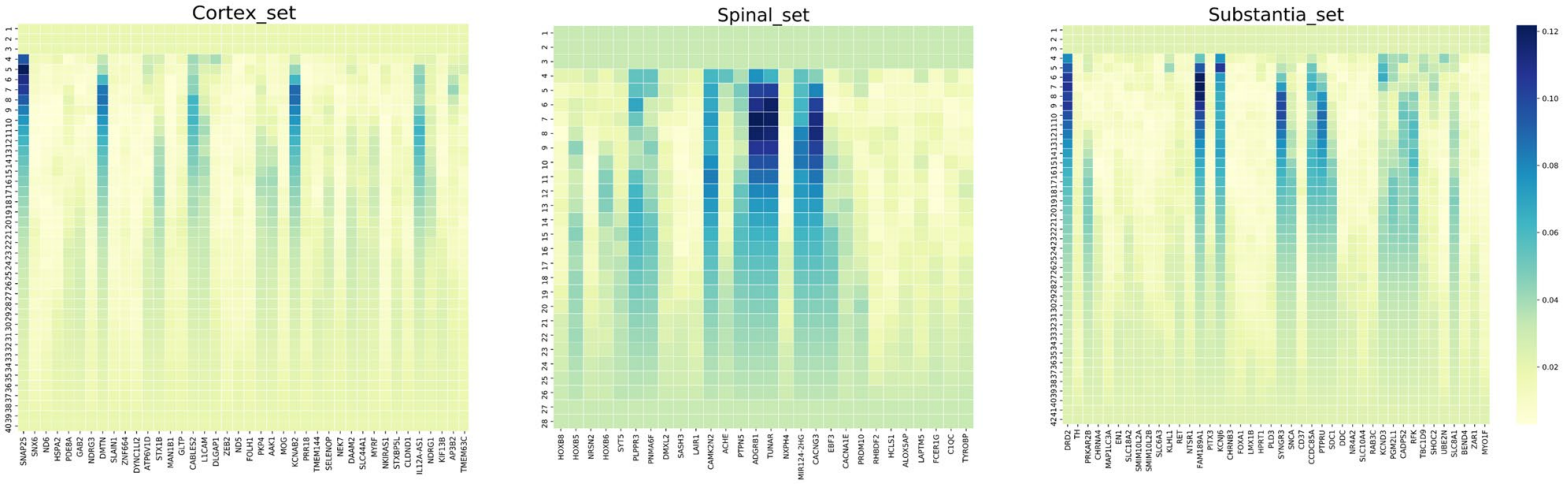
Combinatorial analysis of spinal cord, cortex and substantia nigra gene sets. Heatmaps depicting the frequency of genes present in the classification subsets that were generated at each Gene Oracle Phase 2 iteration. Each row is an iteration and each column is a gene from the cortex/spinal/substantia nigra sets. Darker colors correspond to higher frequencies.

The three smallest significant sets of these five sets were selected for full combinatorial analysis with phase II of Gene Oracle. These included the spinal cord, cortex, and substantia nigra gene sets. Gene Oracle identified the genes which contributed the most to overall classification accuracy (candidate genes) for each of these region-specific sets. Figure 4 contains heatmaps which show the normalized frequency of a gene in a subset at a given iteration of the combinatorial analysis for the spinal cord, cortex and substantia nigra sets. The first three rows of the heatmaps had constant frequency values because all possible combinations of genes were evaluated, hence all genes appeared equivalently in the first three iterations. For the rest of the iterations, the distribution of the frequencies became varied and the most frequent genes appeared. Using the heatmaps, we determined the candidate genes of the three sets to be those with an aggregate frequency of at least one-half the standard deviation above the mean. The rest of the genes were considered “non-candidate” genes. Table 5 shows the candidate genes identified by Gene Oracle for each of these three regions.

**Table 5:** Gene oracle candidate genes for brain GTEx dataset.

Region-specific set	Candidate genes identified by Gene Oracle phase II
Substantia nigra	DRD2, FAM189A1, KCNJ6, SYNGR3, SNCA, CCDC85A, PTPRU, KCND3, CADPS2, RFK, SLC8A1
Cortex	SNAP25, DMTN, STX1B, CABLES2, L1CAM, PKP4, AAK1, KCNAB2, DAAM2, IL12A-AS1
Spinal cord	PLPPR3, CAMK2N2, PTPN5, ADGRB1, TUNAR, MIR124-2HG, CACNG3

Due to computational constraints imposed by the large number of genes, we used a Random Forest approach in lieu of Gene Oracle phase 2 for the hypothalamus and cerebellum sets. We used feature_importances_() built in function to output the most important features (i.e. genes), which were then considered candidates for these two regions. To compare to Gene Oracle, we also ran Random Forest to identify the candidate genes for spinal cord, cortex and substantia nigra. Candidate genes identified by Random Forest are shown in Table 6. The genes denoted in bold are common between the two methods.

**Table 6:** Random Forest candidate genes for brain GTEx dataset.

Region-specific set	Candidate genes identified by Random Forest
Substantia nigra	**DRD2**, TH, EN1, KLHL1, RET, **KCNJ6**, CHRNB3, SDC1, DDC, **CADPS2**, TBC1D9
Cortex	**SNAP25**, **CABLES2**, **L1CAM**, DLGAP1, **PKP4**, **KCNAB2**, NKIRAS1, STXBP5L, **IL12A-AS1**, TMEM63C
Spinal cord	PNMA6F, **CAMK2N2**, **PTPN5**, **TUNAR**, NXPH4, **MIR124-2HG**, EBF3

Genes in bold emphasis are common between the two methods.

**Figure 5:**
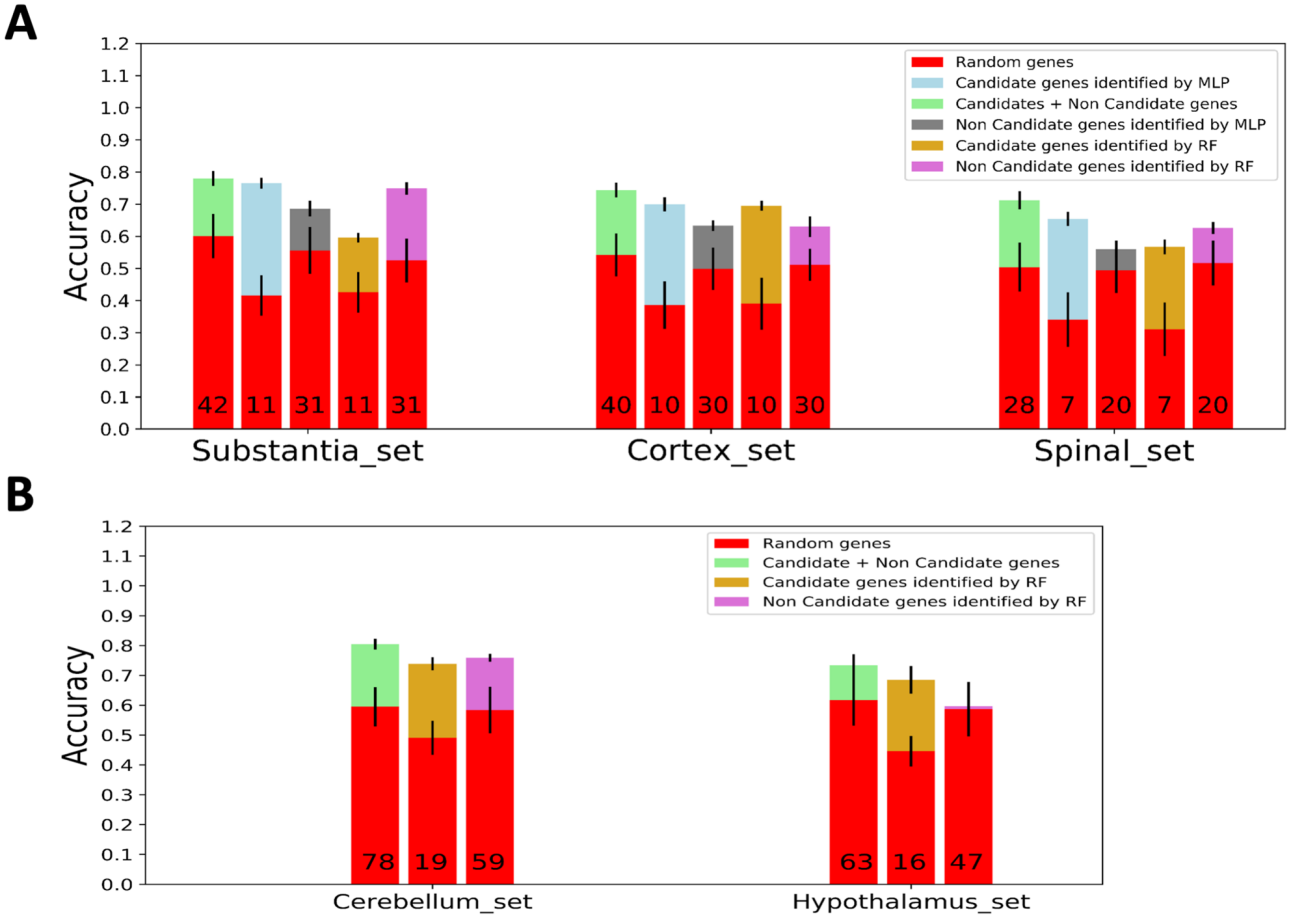
Classification potential for decomposed gene sets. (**A**) Classification accuracies for the full region-specific gene sets (green) were compared to accuracies of the candidate genes identified by Gene Oracle (blue), non-candidate genes identified by Gene Oracle (gray), candidate genes identified by Random Forest (orange), and non-candidate genes identified by Random forest (purple). (**B**) Same as (**A**) but only for decomposed genes identified by Random Forest.

To verify the classification potential for the candidate genes, we ran the Random Forest again for each candidate set shown in Tables 5 and 6. Figure 5 shows, for each region-specific set, the classification accuracy of (1) the original region-specific set, (2) the candidate set identified using Gene Oracle, (3) the non-candidate set identified using Gene Oracle, and to compare to Random Forest, (4) the candidate set identified using Random Forest, (5) the non-candidate set identified using Random Forest. Additionally, the accuracy of each category was compared with the averaged accuracy of 50 random sets of equal size of genes of each set. In all cases, the difference in accuracy from random is highest in the candidate set and the lowest in the non-candidate sets. Furthermore, the candidate set identified by Gene Oracle exhibits a higher difference than those identified by Random Forest as shown in Fig. 5A.

### Brain region biomarker potential for human brain tumors

**Figure 6:**
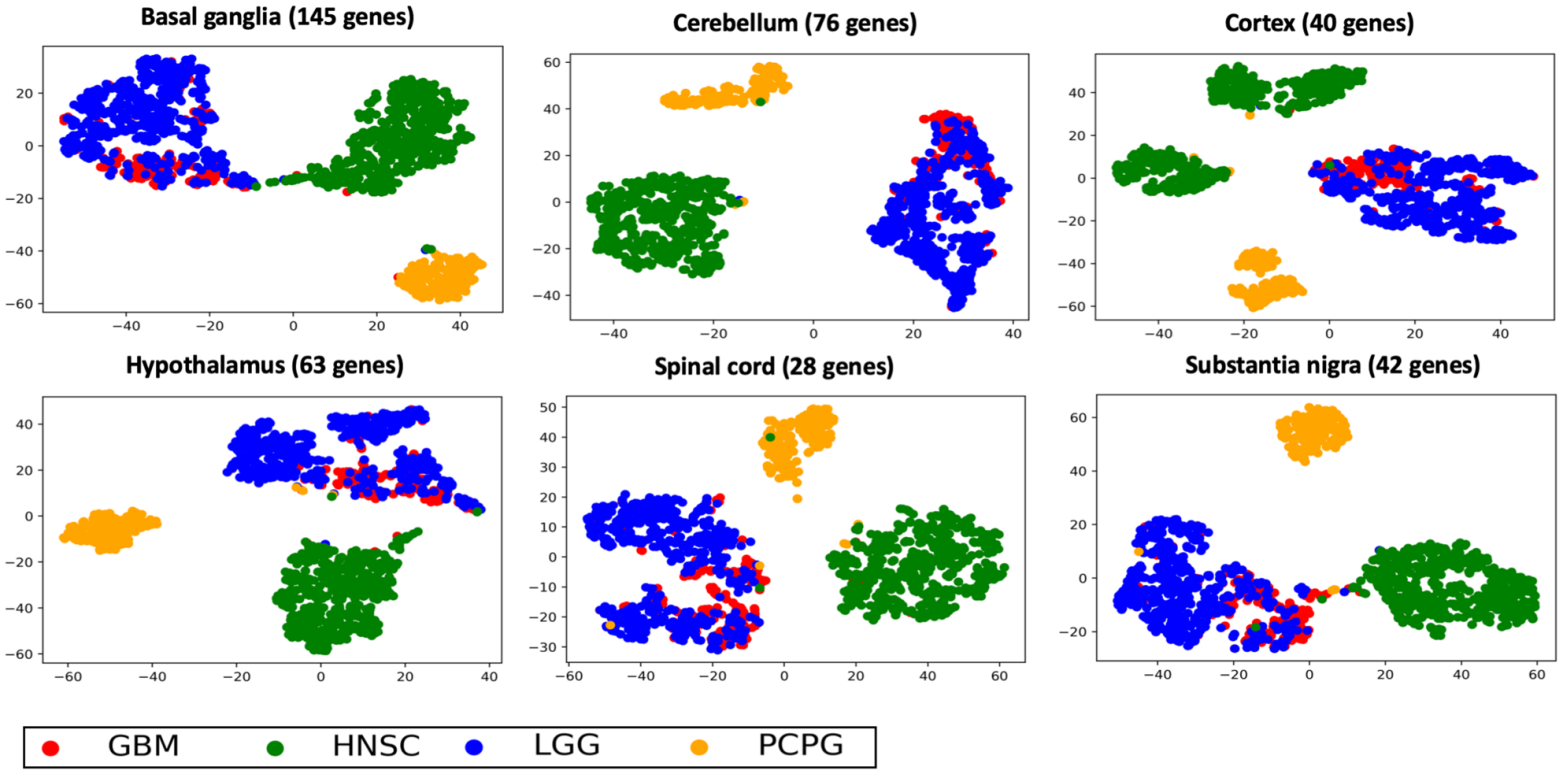
t-SNE visualization of region-specific genes on TCGA tumor data. t-SNE was performed using TCGA RNAseq data from brain region sub-GCN genes. 1431 tumor samples from four tumor subtypes are shown. Tumor RNA expression profiles sorted regions into multiple clusters. Each color represents different regions. Red represents GBM; green represents HNSC; blue represents LGG; yellow represents PCPG.

We were interested to see how the brain region-specific genes could separate abnormal brain samples. For each region-specific gene set, we ran t-SNE on 1,431 tumor samples with four tumor types from The Cancer Genome Atlas (TCGA) as seen in Fig. 6. The tumor types were glioblastoma multiforme (GBM), lower grade glioma (LGG), head and neck squamous cell carcinoma (HNSC), and pheochromocytoma and paraganglioma (PCPG). Most of the region-specific genes could separate HNSC and PCPG tumors apart, while LGG and GBM samples could not be separated. The t-SNE visualization based on 40 cortex specific genes separated HNSC samples and PCPG samples into different sub-groups. For each region-specific tSNE plot, the brain tumors (both LGG and GBM) were separated into 2–3 sub-groups. The t-SNE visualizations of four TCGA subtypes based on enriched nodes for all 13 regions are shown in Supplemental Figure 4. We also ran t-SNE of the 40 cortex specific genes on TCGA tumor data for gender, race and stage. None of these factors could separate tumor clusters (Supplemental Figure 5). tSNE visualization on only brain tumors with IDH mutation annotation is shown in Supplemental Figure 6. For whole brain GCN genes, LGG and GBM were separated apart, but some LGG samples were more similar to GBM samples. The IDH mutant samples were more clearly separated from non-IDH mutant samples. LGG and GBM samples could not be separated using region-specific genes, while IDH mutated samples could be separated with non-IDH mutated samples. IDH mutated samples were also divided into several subgroups using each region’s specific gene list. For example, for tSNE based on substantia nigra specific genes, almost all of the upper samples contain an IDH mutation, while almost all of the bottom samples did not contain an IDH mutation.

In order to see if the brain region-specific genes were important in different tumor types, we aggregated the mutation rates of five TCGA tumor types [GBM, LGG, HNSC, PCPG, and kidney renal clear cell carcinoma (KIRC)] for the six region-specific gene sets and one kidney gene set which contains the 20 most mutated genes in KIRC and their corresponding randomized control genes. As shown in Table 7, the number of mutated genes for all seven gene sets in GBM, LGG and HNSC was significantly higher than that for their corresponding random sets (p-value < 0.01). However, the number of mutated sub-brain specific genes was not significantly higher than the random sets in PCPG tumor. In KIRC tumors, only cerebellum specific gene set were significantly higher in mutated genes than randomly mutated genes. None of the other five region-specific gene sets had significantly numbers of higher mutated genes relative to a similar number of random genes.

**Table 7:** Mutation rates for brain region-specific gene sets in five TCGA tumors.

Region	TS	Polymorphism	Tumor	Mutated	Mutated		TS genes	Random genes		TS genes	Random genes	
	Genes	Detection method	Type	TS genes	Randomized	P-value	Mutated	Mutated	P-value	Total	Total	P-value
					Control gene mean		Tumors	Tumors mean		Mutations	Mutations mean	
Basal ganglia	145	Muse	GBM	122	77.1	< 0.01	139	114.77	0.12	446	308.3	0.04
Basal ganglia	145	Muse	HNSC	124	84.2	< 0.01	286	248.61	0.05	610	496.47	0.09
Basal ganglia	145	Muse	LGG	96	63.6	< 0.01	106	79.55	0.08	389	316.2	0.1
Basal ganglia	145	Muse	PCPG	10	8.7	0.23	11	9.53	0.23	12	9.89	0.21
Basal ganglia	145	Muse	KIRC	67	55.5	0.03	118	91.4	0.04	150	115.49	0.05
Cerebellum	78	Muse	GBM	68	44.9	< 0.01	154	86.21	0.01	402	207.68	0
Cerebellum	78	Muse	HNSC	69	48.4	< 0.01	293	196.33	0	703	344.58	0
Cerebellum	78	Muse	LGG	60	37.8	< 0.01	100	53.93	0.02	428	202.14	0
Cerebellum	78	Muse	PCPG	9	5.48	0.05	10	6.13	0.06	10	6.36	0.07
Cerebellum	78	Muse	KIRC	53	33.39	< 0.01	102	62.69	0	137	73.17	0
Cortex	40	Muse	GBM	34	23.5	< 0.01	49	45.57	0.32	118	103.52	0.26
Cortex	40	Muse	HNSC	36	25.4	< 0.01	126	122.54	0.35	194	176.84	0.26
Cortex	40	Muse	LGG	27	19.4	< 0.01	36	27.44	0.17	129	103.29	0.18
Cortex	40	Muse	PCPG	4	2.45	0.1	4	2.91	0.19	4	2.99	0.2
Cortex	40	Muse	KIRC	19	16.95	0.18	35	34.35	0.36	39	37.99	0.38
Hypothalamus	63	Muse	GBM	60	33	< 0.01	92	54.57	0.04	196	125.23	0.04
Hypothalamus	63	Muse	HNSC	54	36.5	< 0.01	175	135.66	0.04	298	202.4	0.03
Hypothalamus	63	Muse	LGG	44	27.4	< 0.01	56	37.39	0.04	188	134.15	0.06
Hypothalamus	63	Muse	PCPG	2	3.6	0.75	2	3.95	0.78	2	4.14	0.78
Hypothalamus	63	Muse	KIRC	28	22.54	0.01	48	41.54	0.17	55	46.45	0.16
Spinal cord	28	Muse	GBM	20	12.3	< 0.01	33	20.4	0.05	74	41.44	0.02
Spinal cord	28	Muse	HNSC	22	13.5	< 0.01	86	57.66	0.05	113	70.03	0.04
Spinal cord	28	Muse	LGG	21	9.9	< 0.01	22	12.48	0.04	77	42.4	0.02
Spinal cord	28	Muse	PCPG	1	1.1	0.3	1	1.2	0.34	1	1.2	0.34
Spinal cord	28	Muse	KIRC	11	8.21	0.12	19	15.2	0.23	21	16.01	0.17
Substantia nigra	43	Muse	GBM	33	23.4	< 0.01	61	45.25	0.11	123	98.89	0.15
Substantia nigra	43	Muse	HNSC	36	25.4	< 0.01	143	114.3	0.1	198	162.79	0.14
Substantia nigra	43	Muse	LGG	27	19.9	< 0.01	41	28.45	0.05	139	101.72	0.08
Substantia nigra	43	Muse	PCPG	2	2.74	0.5	7	3.2	0.05	8	3.28	0.03
Substantia nigra	43	Muse	KIRC	20	16.76	0.13	30	33.29	0.59	30	36.66	0.7
Kidney	20	Muse	GBM	19	13.44	0.01	18	32.43	0	34	67.57	0
Kidney	20	Muse	HNSC	20	14.07	< 0.01	56	90.82	0	62	121.02	0
Kidney	20	Muse	LGG	19	11.43	< 0.01	8	18.48	0	44	65.24	0
Kidney	20	Muse	PCPG	12	1.8	< 0.01	1	1.96	0	1	1.98	0
Kidney	20	Muse	KIRC	19	10.55	< 0.01	256	25.96	0	507	30.32	0

## Discussion

In this study, we constructed a normal brain GCN and identified edges that were specific to 13 brain regions. After merging edges from similar regions, six brain region-specific GCNs were identified. Functional enrichment for both region-specific modules and the six sub-GCNs provided evidence that these genes encode brain functions. For example, as shown in tissue specific genes functional enrichment analysis in Supplemental Table 6, several edges specific for the substantia nigra were associated with the production of dopamine and other neurotransmitters, as well as their response to amphetamine and nicotine. In addition, both the hypothalamus and basal ganglia specific gene sets are enriched for cilium related functions, such as cilium, cilium movement, motile cilium, and cilium assembly. Cilia play an important role in modulating neurogenesis, cell polarity, axonal guidance and possibly adult neuronal function, which is related to brain development^18^. As expected, the brain GCN encodes brain function.

A prime motivation of our study was to test our approach to identify brain biomarker systems that can distinguish brain regions based upon region-specific co-expression relationships. The idea was that co-expressed genes unique to a brain region would be better biomarkers for sorting samples into normal and aberrant states that involve that region of the brain. Using t-SNE, we visualized the brain region clustering potential of these gene sets. Some gene sets separated regions well (e.g. spinal cord genes, substantia nigra genes, and cerebellum and cerebellar hemisphere genes), while others could not separate samples from each brain region. These visual results suggested that the biomarker sets have varied discriminatory potential.

The six brain region-specific gene sets were also evaluated for quantitative classification potential using a deep learning approach implemented in Gene Oracle to both classify samples from 13 brain regions and identify core candidate gene subsets which play the most important role in brain region classification. Using phase I of Gene Oracle, we examined the classification potential of six gene sets. All sets, except the basal ganglia set, showed significant mean classification accuracy relative to the mean accuracy of the same size of random gene sets (Fig. 3A). The confusion matrix of the basal ganglia gene set, which showed the precise classification and the contribution of each set to each region classification in Fig. 3B, possibly explains why the basal ganglia specific gene set had low classification accuracy. Because the caudate, nucleus accumbens and putamen all belong to the basal ganglia, they are physically located very close to each other. The combined basal ganglia gene set consisted of genes that were only enriched for one of these three regions. However, when we ran Gene Oracle classification, we did not combine the above three regions together. Thus, for example, it is possible that the basal ganglia specific gene sets misclassified caudate into nucleus accumbens or putamen. The same explanation can be applied to the case that most of the sets show a high percentage of misclassification between cerebellar hemisphere and cerebellum. From the confusion plots, we can tell that similar brain regions had the trend to be misclassified with each other, which decreased the classification accuracy. Interestingly, some region-specific gene sets showed the ability to more accurately classify their regions. For example, when the genes of the spinal cord set were used as features, the model was able to classify the spinal cord samples with a 99% accuracy, which is higher when compared to other regions. These results reflect the fact that the region-specific genes can hold a higher predictive power for that region.

We used condition-specific GCN analysis via KINC to identify biomarker candidates. We were able to go one step further using Gene Oracle phase II and Random Forest feature extraction algorithms to identify genes which contributed the most to the overall classification accuracy (candidate genes) for each of the smallest three region-specific sets (substantia nigra, cortex and spinal cord sets). Once compared to the important genes identified by Random Forest, Gene Oracle showed a higher accuracy and a larger increase in accuracy when these genes were used as features (Fig. 5A). For example, the blue box represents the accuracy increase once candidate genes of substantia nigra, cortex, and spinal cord candidate gene sets were used as feature inputs to Gene Oracle. This represents a much higher accuracy compared to the random set (red) and the set identified through the use of Random Forest as a classifier model (orange). More interestingly, Gene Oracle provided deeper resolution than t-SNE. The left panels of Fig. 1B illustrate the large overlap between brain regions whereas Gene Oracle was able to easily discriminate the regions with high accuracy compared to random gene sets. The confusion matrices support this point as well, see Fig. 3B. These results demonstrate the power in utilizing deep learning technology for biomarker gene discovery.

After characterization of the biomarker potential of the brain genes on normal GTEx brain regions, we wanted to test the biomarker systems for classification potential of aberrant brain tissue. For this test, we chose LGG and GBM brain tumors as well as tumors that originated from other organs. t-SNE visualization was performed using TCGA tumor RNAseq expression profiles for the brain genes on samples from four different tumor types. Most of the region-specific genes could separate HNSC and PCPG tumors while LGG and GBM samples could not be separated. Interestingly, even though LGG and GBM samples could not be separated apart, those tumor samples can still be separated into several subgroups. This is partially due to the status of the IDH1/2 mutation which is very common in LGG^19^ (Supplemental Figure 6). The IDH mutant gliomas can be further divided into smaller sub-groups as well. Moreover, the t-SNE plots showed that the 40 cortex specific genes separated HNSC samples and PCPG samples into different sub-groups. As with the normal brain samples, there was mixed potential of the genes to sort human tumors.

Interestingly, we found that many of the brain region-specific GCN edges were mutated in tumors. As shown in Table 7, the number of mutated genes in all six brain region-specific gene sets was significantly higher than the number of mutated genes in the list of size-controlled random genes for GBM, LGG, and HNSC (p-value less than 0.01), but not for PCPG and KIRC. GBM and LGG represent tumors that originate in the brain. HNSC is not a brain cancer, but it originates in the squamous cells that line the moist, mucosal surfaces inside the head and neck, such as mouth, nose, throat, larynx, sinuses, or salivary glands^20^. PCPG originates mainly on the adrenal gland and only a few cases of paraganglioma localize in the neck and head^21^. KIRC originates from the kidney. The brain region-specific gene sets had a higher mutation rate in the brain tumors (LGG and GBM) than other tumors when compared to random genes. Interestingly, all brain region-specific gene sets had significantly higher mutation rate than random genes in HNSC, which indicates that these six brain specific gene sets could be important in HNSC tumor formation. Furthermore, cerebellum specific gene sets showed significantly higher mutation rate for KIRC, which means these 78 cerebellum specific genes may also play an important role in KIRC formation and development. The kidney specific gene set had higher mutation rates in almost all five listed tumor types. This might be because we chose the top 20 most mutated genes in KIRC as identified in the TCGA data portal. The 20 chosen genes are not necessarily specific to kidney tumors, and therefore could also be highly mutated in other tumors. Thus, these genes that are mutated in KIRC also have significantly high mutation rates in HNSC, LGG and PCPG.

In conclusion, this study describes how condition-specific candidate biomarker systems can be discovered using GCN analysis and we describe how machine learning approaches can be used to measure the quality of the biomarker sets. Further, we believe that the significant condition-specific relationships are worthy of deeper analysis into why they are present in specific brain regions. In the future we intend to further investigate the biological significance of these edges, including an examination of normal region eQTL regulation of these gene datasets to find important transcription factors and binding sites that may become altered during tumorigenesis.

## Methods

### Input data and gene expression matrix (GEM) preparation

All available gene-level TPM (transcripts per million) files for 13 normal brain region samples were downloaded from the Genotype-Tissue Expression (GTEx) project version 7^10^. 1671 samples were downloaded–each containing measurements of 56,202 genes—and merged into a GEM. The matrix underwent preprocessing steps, including log base 2 transformation, quality control, and quantile normalization, using the preprocessCore R library^22^. The Kolmogorov–Smirnov test (KS Dval> 0.15)^23^ was performed to test for outlier samples. No samples qualified as an outlier, so we continued with a quantile normalization of the matrix to reduce any technical noise. All FPKM (fragments per kilobase of gene per million read pairs) files for GBM, LGG, HNSC, and PCPG patients were downloaded from TCGA using the GDC Data Transfer Tool^24^. 1431 samples with 60,483 genes were aggregated into a GEM. The GEM underwent the same preprocessing steps as the GTEx GEM.

### Gene co-expression network construction

KINC (https://github.com/SystemsGenetics/KINC) was used to identify gene correlation relationships within the normalized GTEx brain GEM. The algorithm calculates correlation for each gene pair after clustering samples using GMMs. Only clusters with equal to or more than 30 samples underwent Spearman correlation. We submitted 50,000 KINC similarity jobs on the Open Science Grid^25^ by using the OSG-KINC similarity workflow^26^. The workflow was accomplished using the Pegasus Workflow manager^27^. Normalized TPM expression values less than 0 were ignored. KINC similarity output was transferred to Clemson University’s Palmetto Cluster via Globus. The KINC significance threshold of 0.8961 was found by using a random matrix thresholding (RMT) algorithm within the KINC thresholding script. The GTEx brain GCN was then constructed by extracting all edges with correlations> 0.8961 using the KINC extract script. 183 linked community modules (LCMs) were identified by the linkcomm R packages with a minimum cluster size of 3 edges^16^. The full GCN is shown in Supplemental Table 1.

### Edge and module sample enrichment analysis

All identified modules and edges in the GTEx brain GCN were tested for sample label enrichment using the KINC.R package (https://github.com/SystemsGenetics/KINC.R). A Fisher’s exact test with a Hochberg p-value correction was used as the default arguments to the analyzeNetCat function. The sample label enrichment lists for modules as well as edges are shown in Supplemental Table 2 and 3. In the sample label enrichment for modules, we considered to use the p-value threshold of 1E−3 for significantly enriched modules. The edges with p-value less than 1E−10 were considered as significantly enriched edges because this p-value is close to maximizing the number of edges and nodes. The number of enriched edges for each specific region with the adjusted p-value less than 1E−3, 1E−5, 1E−15, and 1E−20 were also collected separately and the tSNE visualization was run for each of those gene sets (Supplemental Table 4 and Supplemental Figure 1). Furthermore, we calculated the number of regions that each edge, module, and eQTL belonged to. eQTL datasets for 13 brain regions samples were also downloaded from the GTEx project V7^10^. region-specific edges and modules were selected to construct GTEx sub-brain GCNs. LCM modules were also tested for functional term enrichment using the FUNC-E package (https://github.com/SystemsGenetics/FUNC-E), which uses a Fisher’s exact test similar to the David^28^ method of functional enrichment. For cross-module comparisons, enriched terms were considered significant if the Bonferroni-corrected p-value was less than 0.001. Functional annotations performed include Gene Ontology^29^, Reactome^30^, Pfam^31^, Interpro^32^, and Mendelian Inheritance in Man (MIM)^33^. The full module functional enrichment list is shown in Supplemental Table 5. region-specific edges for each region also underwent functional term enrichment analysis, which is shown in Supplemental Table 6. Moreover, gene type for all currently identified genes was downloaded from Ensembl Biomart (https://useast.ensembl.org/info/data/biomart/). All genes from GTEx dataset, genes from brain GCN as well as each region-specific genes were counted for calculating the protein coding and non-coding gene percentages. This result is shown in Supplemental Table 7 and Supplemental Table 8.

### t-SNE analysis

A dimensionality reduction and visualization pipeline was performed using either a full or partial GTEx brain GEM as the input. This allowed us to compare how varying subsets of genes were able to separate the selected brain regions. It was performed using the principal component analysis (PCA) and t-distributed stochastic neighbor embedding (t-SNE) Python sklearn packages^17^. Each t-SNE run created a two-dimensional randomly initialized embedding, in which samples were clustered into different sub-groups. The perplexity used for each run was 30. This pipeline was performed on the GTEx brain GCN GEM containing 1691 genes for 1671 samples, as well as the six GTEx sub-brain GCN GEMs containing region-specific genes for 1671 samples. We also performed PCA and t-SNE on the TCGA cancer GEM, which contained region-specific genes for 1431 tumor samples, in order to segregate the four TCGA tumor types. TCGA datasets were downloaded from TCGA data portal^34^.

### Brian region classification

We used a two phase, bottom-up classification approach of a feedforward neural network, known as Gene Oracle^15^ (https://github.com/SystemsGenetics/gene-oracle), to classify brain regions, and thus, identify the region-specific gene biomarkers. Gene Oracle uses a multilayer perceptron (MLP) feedforward neural network^35^ to identify biomarker gene sets with a significant classification accuracy when comparing to sets with equal number of random genes. Gene Oracle can also sort genes within a gene set according to their classification rates. This is done by breaking the gene set down into its most discriminatory features, followed by iteratively appending genes to explore new combinations. The architecture of the network consists of a total of five layers: an input layer with a size equivalent to the size of the gene set, three hidden layers (512, 256, and 128 units, respectively), and a final layer for classification. The three hidden layers utilize rectified linear unit (ReLU) activation function^36^. In Gene Oracle phase I, six merged brain region gene sets were screened for a significant classification potential that would allow for classification of the samples into 13 brain regions. For each brain gene set, 50 random size-controlled gene sets were selected from all genes in the input GTEx GEM and evaluated using the same classifier. Size-control means that each corresponding gene in the random list was within 10% of the size of the original gene from the region-specific list. The mean classification accuracy was calculated for the 50 random gene sets and compared with the corresponding brain gene set accuracy. For example, the cortex set that contained 40 genes was compared to 50 different sets of 40 random size-controlled genes for classification accuracy. A 10-fold cross validation procedure was applied to train and test the model. A gene set was chosen to undergo further analysis if the classification accuracy was higher than that of the average of the corresponding random sets with a statistical significance of p < 0.001 (using Student’s *t* test). In Gene Oracle phase II, the gene set that exhibited a significant classification potential underwent a combinatorial decomposition in order to discover the most discriminatory genes in the set. Three brain gene sets with a smallest number of genes, including the cortex gene set, the spinal cord gene set, and the substantia nigra gene set, underwent Gene Oracle phase II to detect candidate genes for better classification.

To compare the results of Gene Oracle phase II, we utilized Random Forest^37^ to run the classification for the five brain sets that were significant in Phase I of Gene Oracle. Random Forest was also used to highlight the important features (i.e. genes) using its built in functions of scikit-learn library in Python. Once Random Forest identified the important features, they were compared to the ones identified by Gene Oracle. The Random Forest model consisted of 100 trees, where the value of the threshold for early stopping in tree growth is 1E−7. The built-in scikit-learn function “RandomForestClassifier” was used to construct the Random Forest model in Python.

### Tumor gene mutation rates

Somatic mutations for GBM, LGG, HNSC, PCPG, and KIRC tumor subtypes were downloaded from TCGA^34^. TCGA reported mutations from four different polymorphism detection methods including Muse^38^, Mutect^39^, Sniper^40^ and Varscan^41^. We downloaded the Muse method dataset and counted the number of tumors with at least one mutation, the number of genes mutated in a tumor, and the number of total mutations present in a tumor. We summed these values for candidate gene sets and the size-controlled random gene sets of equal number. The randomized control genes were counted a hundred times and then an empirical p-value (p < 0.01) was determined for each candidate gene set. The absence or presence of an IDH mutation (IDH1/IDH2/IDH3) in LGG and GBM samples was also collected and used for tSNE visualization.

## Supplementary information

Supplementary Figures.

Supplementary Tables.
