# Supplementary material for: Exploration into biomarker potential of region-specific brain gene co-expression networks
Source: Sci Rep. 2020 Oct 13;10:17089. doi: 10.1038/s41598-020-73611-1 (PMC7553962; doi:10.1038/s41598-020-73611-1)
Supplement: Supplementary file 1 — Supplementary Figures. [file 41598_2020_73611_MOESM1_ESM.docx]

**Supplementary Figures**

**Exploration into Biomarker Potential of Region-Specific Brain Gene Co-expression Networks**

Yuqing Hang^1^, Mohammed Aburidi^2^, William L. Poehlman^1^, Benafsh Husain^2^, Allison Hickman^1^, and F. Alex Feltus^1,2,3,*^

^1^Clemson University Department of Genetics & Biochemistry 29634 USA

^2^Clemson University Biomedical Data Science and Informatics Program 29634 USA

^3^Clemson University Center for Human Genetics 29634 USA

*Corresponding Author. Email: [ffeltus@clemson.edu](mailto:ffeltus@clemson.edu)

**
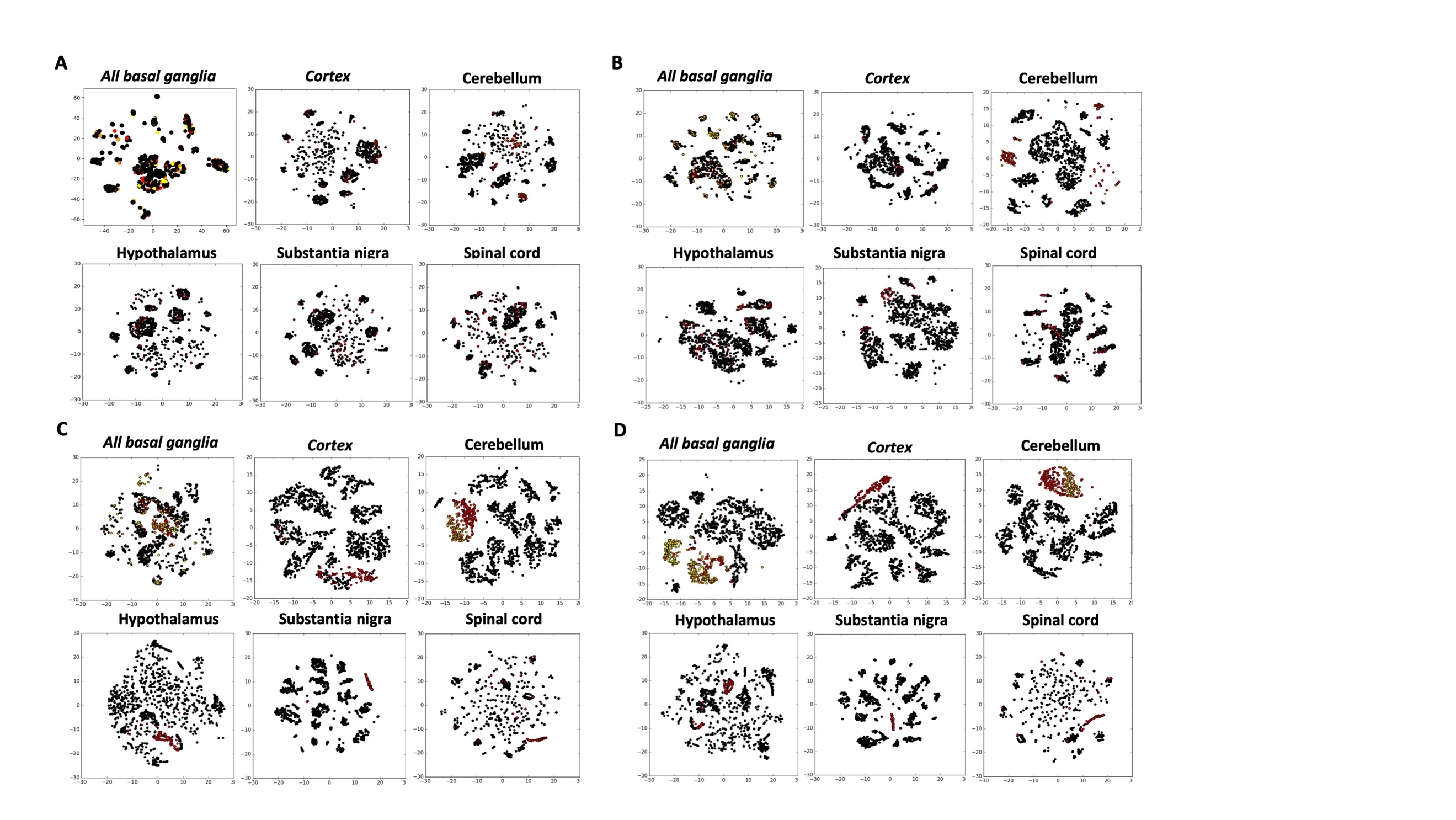
**

**Supplemental Figure 1. tSNE visualization based on region specific genes with different p-values.** Figure (A), (B), (C), (D) represents the genes with p-value less than 1.0e-3 1.0e-5, 1.0e-15, 1.0e-20 respectively. Non-black dots represents the tissue subtypes represented by the mini-GCN and black dots represents all other tissue types. For all basal ganglia specific gene sets, red, orange and yellow dots represents caudate basal ganglia, nucleus accumbent basal ganglia, and putamen basal ganglia samples respectively. The red and orange dots from cerebellum and cerebellar hemisphere specific gene sets represent cerebellum and cerebellar hemisphere samples respectively. All red dots from other tissue specific gene sets represent the particular tissue specific samples.

**
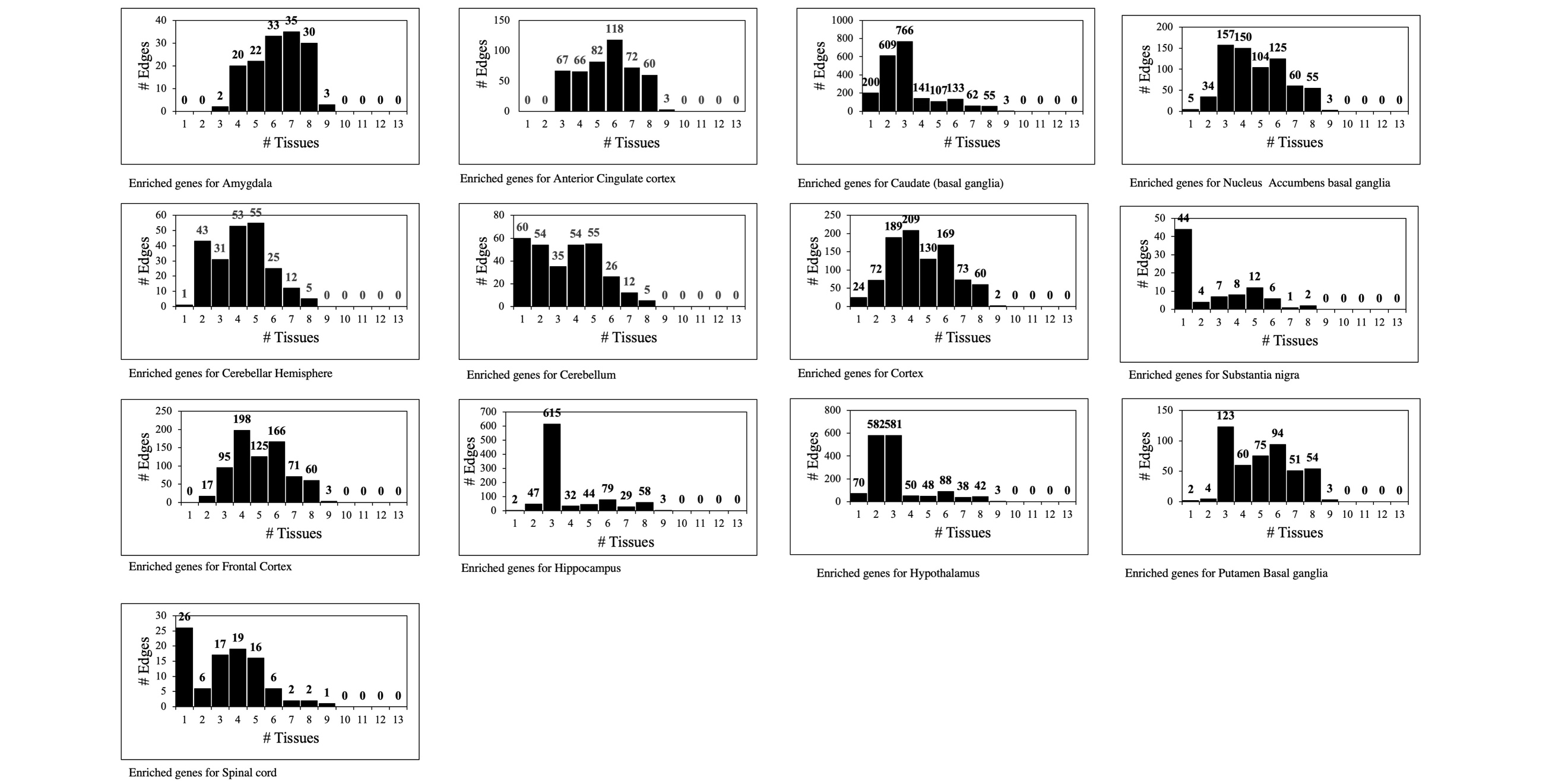
Supplemental Figure 2. Number of edges unique to 1 to 13 different brain regions for each region.**


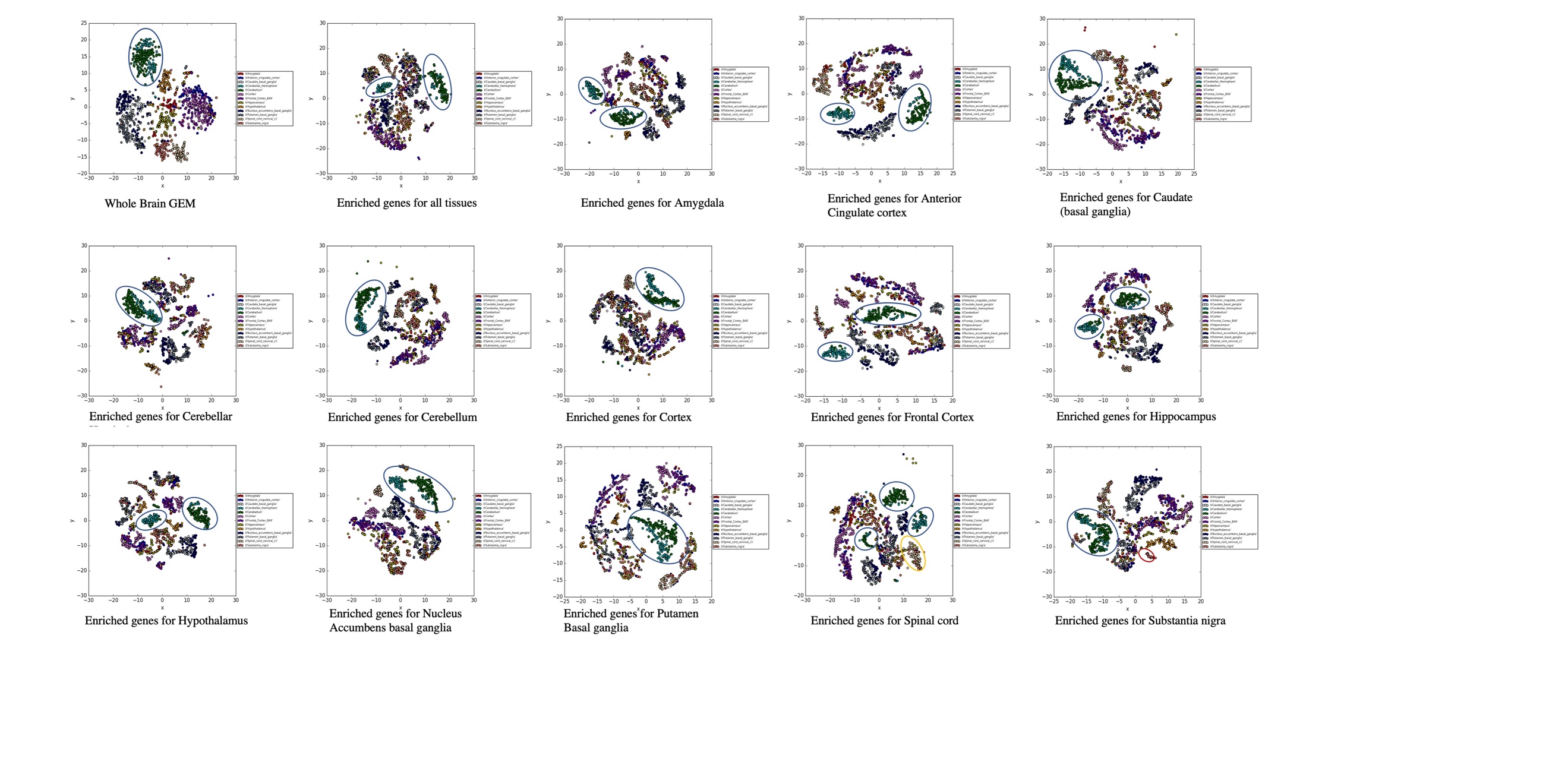


**Supplemental Figure 3. tSNE based on gene expression of enriched nodes for each region with p-value at 1.0e-10.** For each tSNE plot, the different colors represent different regions. The first picture represents tSNE visualization from all brain genes that collected from brain GCN. Second picture is the tSNE results from total number of genes that are enriched for any of the 13 brain regions. The next 13 figures represent the gene sets for the specific region. The blue circle represents cerebellum and cerebellar hemisphere samples, yellow circle represents spinal cord samples, and red circle represents substantia nigra samples.

**
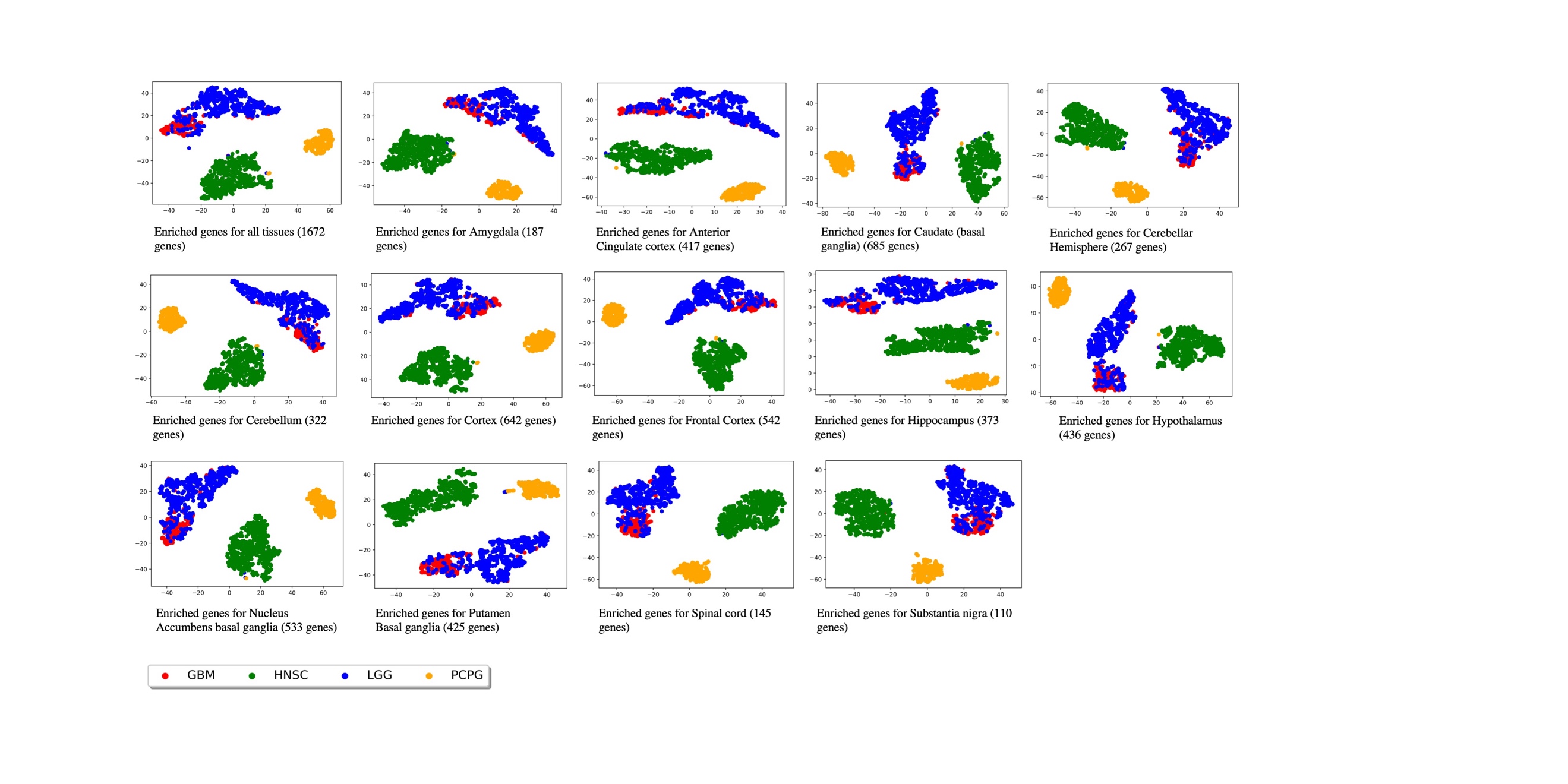
**

**Supplemental Figure 4. TCGA 4 tumor types tSNE based on gene expression of enriched nodes for each region.** t-SNE was performed using TCGA RNAseq data from brain region enriched genes. These genes can be enriched in more than one region. 1431 tumor samples from four tumor subtypes are shown. Tumor RNA expression profiles sorted regions into multiple clusters. Each color represents different regions. Red represents glioblastoma (GBM); green represents head and neck squamous cell carcinoma (HNSC); blue represents low grade glioma (LGG); yellow represents pheochromocytoma and paraganglioma (PCPG).

**Supplemental Figure 5. tSNE visualization of cortex specific genes on TCGA tumor data for gender, race, and stage.** tSNE embedding of the 1424 tumor samples of 4 tumor types x each corresponding region specific gene subsets. RNA expression profiles sorted regions into multiple clusters. Different color represents different region. (A) gender perspective: Red represents male; blue represents female. (B) race perspective: red represents white people; blue represents black or African American; orange represents Asian; yellow represents American Indian or Alaska native; green represents Not reported. (C) stage perspective: red represents stage i; blue represents stage ii; orange represents stage iii; yellow represents stage iva; green represents stage ivb; cyan represents stage ivc; black represents Not reported.

A

B


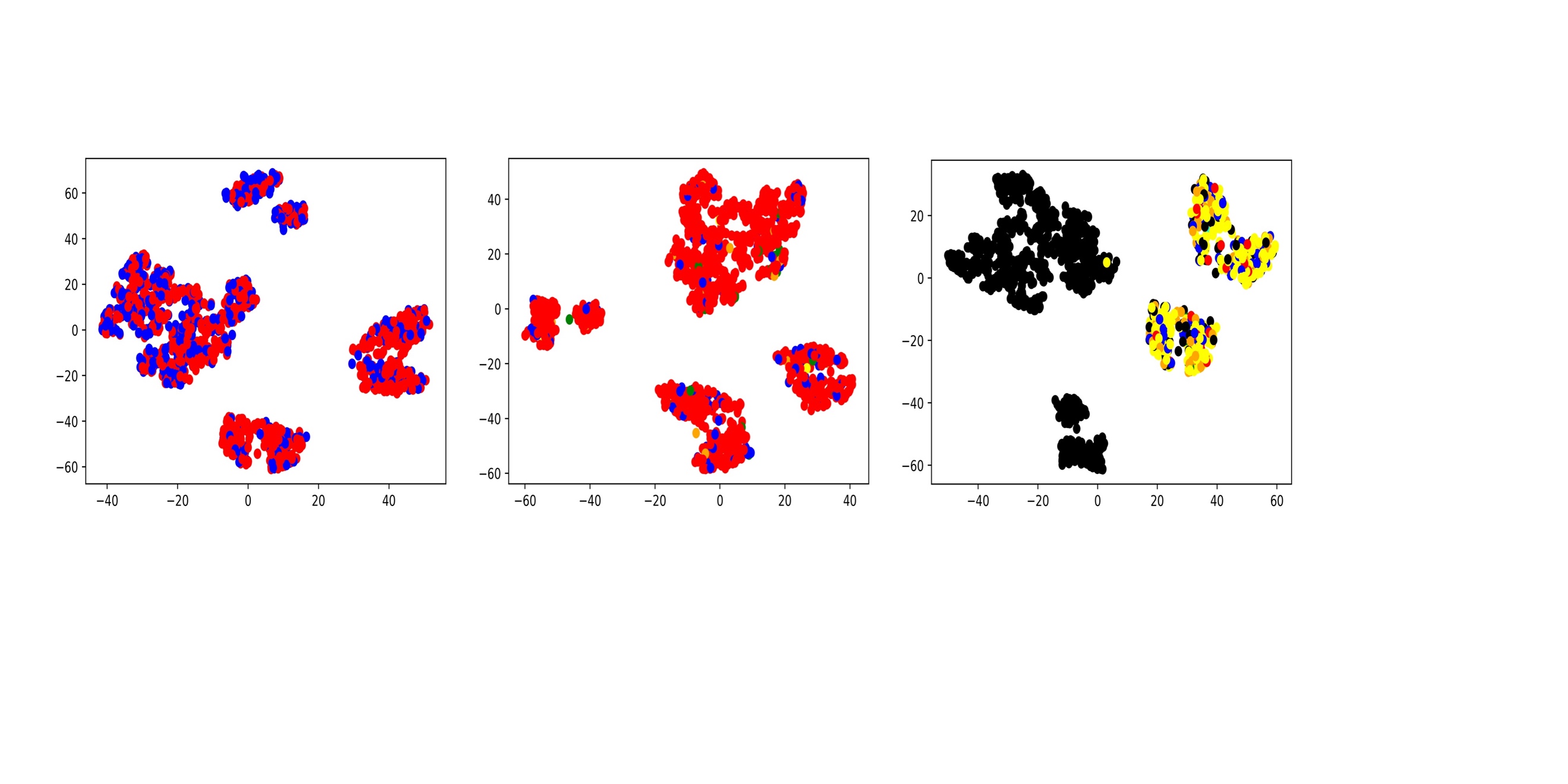


C

**
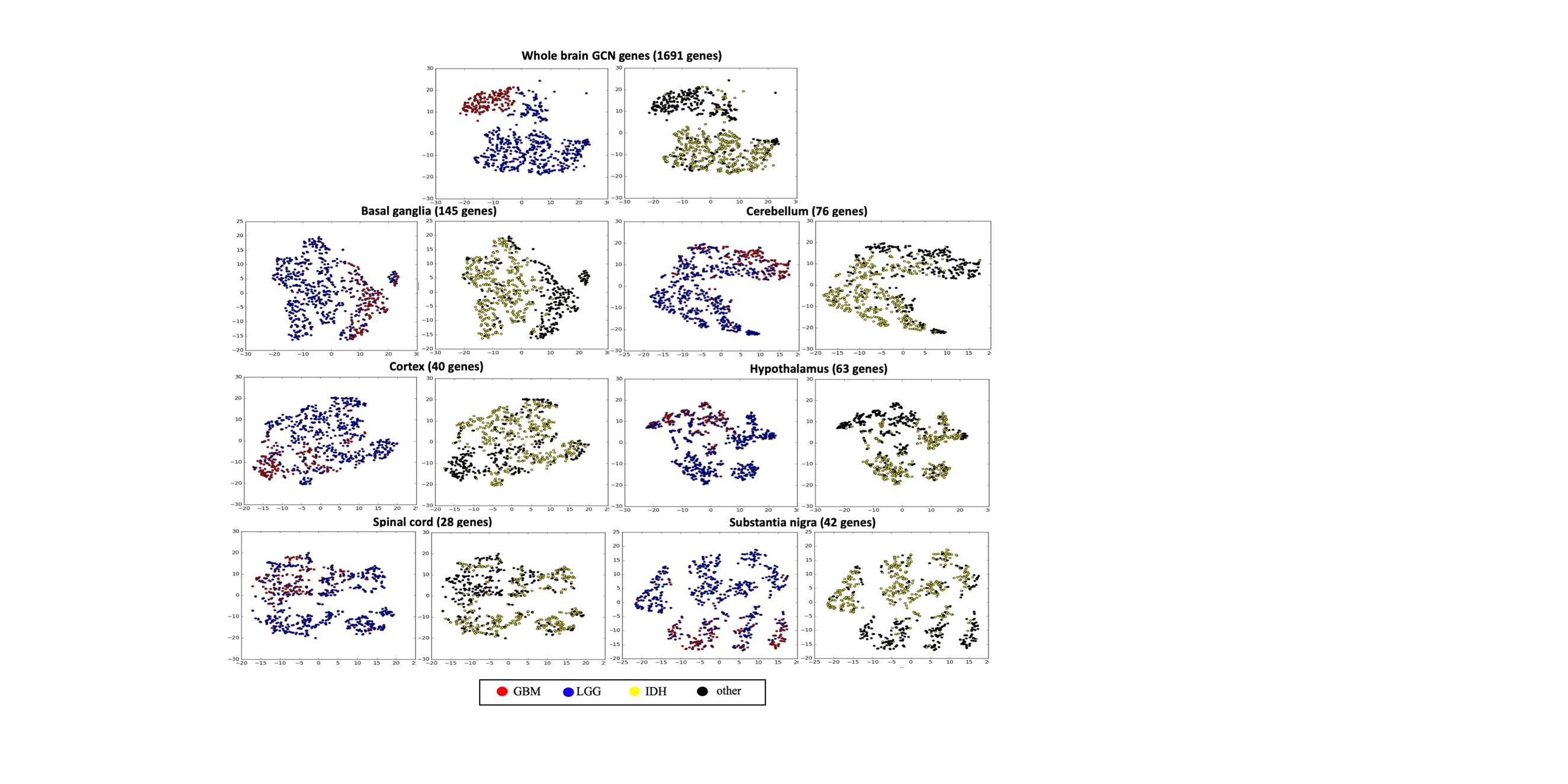
Supplemental Figure 6. tSNE visualization of region specific genes for brain tumors based on IDH mutation.** The first two pictures are tSNE plots from whole brain GCN genes. The bottom six tSNE plot pairs are based on brain region sub-GCN genes. For each pair, left picture is tSNE plots annotated by tumor type, while right panel is annotated by the samples whether contain IDH mutation.
